# Classification of Emotional Expressions Is Affected by Inversion: Behavioral and Electrophysiological Evidence

**DOI:** 10.3389/fnbeh.2017.00021

**Published:** 2017-02-09

**Authors:** Jian Song, Min Liu, Shun Yao, Yan Yan, Huichao Ding, Tianyi Yan, Lun Zhao, Guozheng Xu

**Affiliations:** ^1^Department of Neurosurgery, Wuhan General Hospital of PLAWuhan, China; ^2^No 457 Hospital of PLAWuhan, China; ^3^Brain Research Institute, Beijing Yiran Sunny Technology Co. Ltd.Beijing, China

**Keywords:** face classification, expression, face inversion, N170

## Abstract

It has been shown that emotionally positive facial expressions are recognized substantially faster than emotionally negative facial expressions, the positive classification advantage (PCA). In this experiment we explored the involvement of configural computations while processing positive and negative faces in an expression categorization task using artificial faces. Analyzing the reaction times (RTs), we found that happy faces were categorized more quickly than sad faces (PCA) and this effect disappeared for inverted faces. Event-related potentials (ERPs) data showed that the face-sensitive N170 component was larger for sad than for happy faces only at upright condition and that face inversion significantly enhanced N170 amplitudes only for happy faces. Moreover, the happy faces elicited shorter N170 latency than did the sad faces, whereas for inverted condition the N170 latency did not differ between happy and sad faces. Finally, the significant positive correlation between the RTs and the latency of the N170 was not found for N170 amplitudes. Because the configural computation was task-irrelevant in the present study, these behavioral and ERP data indicated that one of the sources of PCA is the configural analysis applied by default while categorizing facial emotions.

## Introduction

Facial expressions reflect a person’s emotional state, current motives and intentions. It is therefore important for adaptive purposes that the cognitive system can rapidly extract accurate information from the observed expressions. It has been shown that emotionally positive facial expressions are recognized substantially faster than negative facial expressions, the positive classification advantage (PCA). This effect was conspicuous for happiness recognition faster than sadness (e.g., Crews and Harrison, 1994; Leppänen and Hietanen, 2004; Liu et al., 2013), anger (e.g., Billings et al., 1993), disgust (e.g., Stalans and Wedding, 1985) and emotional neutrality (e.g., Hugdahl et al., 1993; Liu et al., 2013).

Several perceptual strategies are used by humans while processing faces: local and configural processing (e.g., Maurer et al., 2002). Local information mostly refers to distinct circumscribed characteristics of the face, such as the mouth or the nose. General spatial relations of the face (e.g., the eyes are above the nose) are usually described as configural information or first-order relations, whereas second-order relations refer to specific spatial relations (e.g., distance between eyes and nose) and possess a higher discriminative value (Leder and Carbon, 2006). Evidence has accumulated that the configural analysis underlying face recognition also applies to facial-emotion recognition, being dependent upon facial features and spatial arrays (e.g., McKelvie, 1995; Calder et al., 2000). For example, McKelvie (1995) assessed the effect of face inversion on the recognition of facial expressions of emotion and found that inversion impaired the recognition of sad, fearful, angry and disgusted, but not of happy expressions.

Face classification is based on visual information that is similar to all “facial action patterns” irrespective of the faces that are making them and the expression classification processes of faces include the extraction of attributes of expressions (Ganel and Goshen-Gottstein, 2002). Although the PCA has been proposed in previous studies, it is less clear whether configural processing is also required in the classification of facial expressions of emotion. The present research was designed to address this issue by recording the N170 of event-related potentials (ERPs) while the participants categorized the upright and inverted face stimuli according to their expressions.

The N170 component at occipito-temporal electrodes, a negative ERP occurring between 140 ms and 180 ms after stimulus onset, is the earliest component associated with face perceptual processing and is reliably larger to faces than other stimulus categories (Bentin et al., 1996). Based on data showing that the N170 is not sensitive to the face identity (Bentin and Deouell, 2000; Eimer, 2000; Anaki et al., 2007), larger (and delayed) for face components (particularly eyes) than full faces (Bentin et al., 1996; Itier et al., 2006), larger (and delayed) for inverted faces (Bentin et al., 1996; Rossion and Gauthier, 2002) and equally large for scrambled and normally configured faces (Zion-Golumbic and Bentin, 2007), it was suggested that the N170 is closely relevant to the detection of global face structures as well as other information of faces. Importantly, several studies found that the N170 component was entirely unaffected by any of the basic emotional expressions (e.g., Eimer and Holmes, 2002; Ashley et al., 2003; Eimer et al., 2003), implicating that expression processing of faces occurs at post-perceptual stage. Recently, however, growing evidence suggests that the N170 can be modulated by facial emotion, e.g., happy faces elicit smaller amplitude than other emotions (e.g., Caharel et al., 2005). One recent study investigated the time course of the PCA by recording ERPs and found that, compared with sad faces, happy faces elicited a smaller N170 (Liu et al., 2013). However, in Liu et al. (2013) study the face inversion effect was not investigated.

The goal of the current study was to map the effect of face inversion on the early stage of face classification by expression. In the present study, we adopted schematic face stimuli like previous studies (e.g., Leppänen and Hietanen, 2004; Liu et al., 2013). Several studies using schematic facial expressions, emoticons or smileys have shown the comparable emotional effect elicited by photographic facial expressions (Boucsein et al., 2001; Eger et al., 2003; Babiloni et al., 2010). Schematic faces may be ideal experimental stimuli because they allowed us to fully control the low-level physical features, to exclude additional information related to facial identity, such as gender, race, etc., and to minimize the confounding effects of general arousal rather than valence *per se*. As the specific index of configural processing, face inversion disrupts the global configural information, resulting in the decrease of recognition accuracy, the increase of reaction time (RT) and the enlargement or delay of N170 component. If the PCA phenomenon relies more on high-level configural information, it is expected attenuated PCA in face inversion condition.

## Materials and Methods

### Participants

Thirty-six young healthy individuals participated in our study (16 female, aged 20–25 years, mean age: 22.6 years). All participants were right-handed and had normal or corrected-to-normal visual acuity and were free of a neurological or psychiatric history. They received payments for their participation and gave their written informed consent before the experiment. This study was approved by the Ethical Committee of Wuhan General Hospital in accordance with the ethical principles of Declaration of Helsinki.

### Stimuli

**Figure 1:**
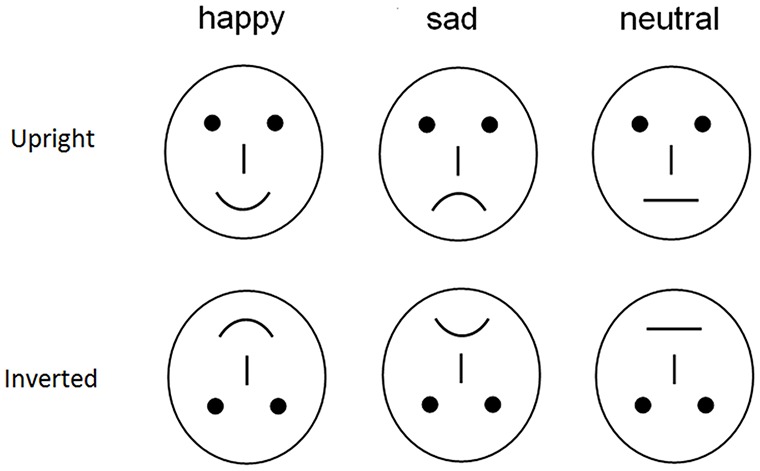
**Examples stimuli of facial expression**.

To avoid the low-level processing of facial features as well as boredom by the excessive repetition of one single model, each emotional category consisted of 20 different schematic face models by manipulating the distance among facial features and by manipulating the shape of the facial features (Figure 1; Liu et al., 2013). All stimuli were presented at the center of a video monitor and viewed from a distance of 100 cm at a visual angle of approximately 7.27 × 6.06°. The experiment consisted of four blocks of 120 trials each (480 trials in total with 80 trials × 3 expressions × 2 orientations).

### Procedure

Following the electrode application, the participants were seated in a dimly lit and sound-attenuated cabin. They were instructed to classify each face by the expression it represented and to respond to sad or happy faces (ignoring neutral faces) by pressing correspondingly labeled buttons on the keyboard with the left index finger (“Z” key) or right index finger (“/” key), respectively. Speed and accuracy were equally emphasized. All 480 stimuli were randomly presented in a mixed design, with four blocks of 120 stimuli each, with a short break in between, and the labels of the response buttons (happy–sad/sad–happy) were counterbalanced across the participants. Each face was presented for 300 ms with an intertrial interval ranging randomly between 600 ms and 800 ms, starting after response. The participants completed one practice sequence of 30 stimuli (five from each type, equally representing the three facial expressions). These stimuli were not used in the main experiment.

### EEG Recording

EEG was recorded continuously by Neuro-Cap with a set of 32 Ag/AgCl electrodes placed according to the 10/20 system. In order to monitor eye movements and blinks, EOG was recorded via electrodes placed on the bilateral external canthi and the left infraorbital and supraorbital areas. Both EEG and EOG signals were sampled at 500 Hz, with a 0.1–100 Hz band pass using a NeuroLab^®^ digital amplifiers system. During recording, we used the tip of the nose as reference and a common average reference was calculated off-line. Electrode impedances were kept below 5 kΩ.

We corrected EOG artifacts off-line using a correlation method proposed by Semlitsch et al. (1986) and supplied as part of the EEGLab software. The EEG was segmented in epochs of 1000 ms beginning 200 ms prior to stimulus onset and averaged separately for each condition (happy and sad faces for upright and inverted conditions, respectively). Segments with an incorrect response or contaminated with peak-to-peak deflection exceeding ±100 μV were excluded from averaging. After this procedure, averaged ERPs included at least 65 trials for each of face conditions. The averaged ERP waveforms were low-pass filtered at 30 Hz (24 dB/octave).

### Data Analysis

RTs (from the stimulus onset) and accuracy rates were recorded and analyzed using a two-way analysis of variance (ANOVA) with *Expression* (happy, sad) and *Orientation* (upright, inverted) as within-subject factors.

**Figure 2:**
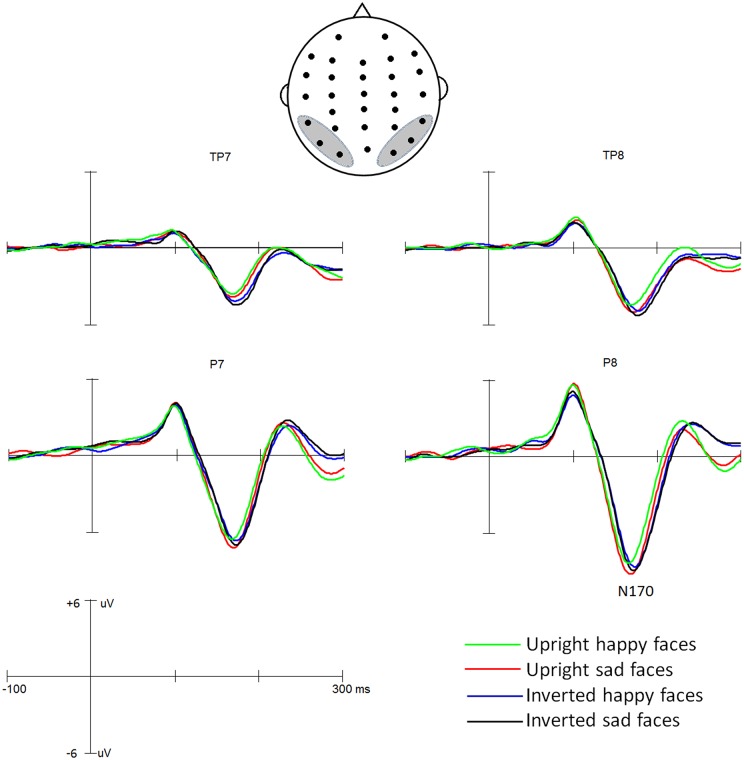
**The N170 for facial expression in upright and inverted orientations**.

Based on previous studies (e.g., Bentin et al., 1996) and limited by the 32-sites montage (see montage in Figure 2), the peak amplitudes and latencies of the N170 were measured automatically between 120 ms and 200 ms at P7, P8, TP7, TP8, O1 and O2 sites. These measures were analyzed using a four-way ANOVA with *Expression* (happy, sad), *Orientation* (upright, inverted), *Hemisphere* (left, right) and *Site* (P7/8, TP7/8, O1/2) as within-subject factors. Degrees of freedom were corrected whenever necessary using the Greenhouse–Geisser epsilon correction factor.

## Results

### Performance

A 2 (*Expression*) × 2 (*Orientation*) repeated-measures ANOVA was conducted for the percentage of correct responses. Neither the main effect of *Expression* (93.5% and 93.0% for happy and sad faces, respectively; *F* < 1) nor the main effect of *Orientation* (93.3% and 93.2% for upright and inverted faces, respectively; *F* < 1) was significant. The two-way interaction was not significant (*F* < 1).

For each participant, incorrect responses or responses with RTs more than ± 2 *SD*s from the mean in each condition were excluded for RT analysis. On average, 8.7% of responses were removed (Table 1). The RTs were analyzed using the same statistical model as that for percentages of correct responses. There was a significant main effect of *Expression, F*_(1,35)_ = 7.49, *p < 0*.01, partial *η*^2^ = 0.176, showing that happy face categorization was faster (598 ms) than classifying sad faces (615 ms). The main effect of *Orientation* was also significant, *F*_(1,35)_ = 146.6, *p* < 0.001, partial *η*^2^ = 0.807, showing that upright faces was classified more quickly (584 ms) than classifying inverted faces (629 ms). Importantly, we found the significant two-way interaction of *Expression* * *Orientation, F*_(1,35)_ = 9.45, *p* < 0.01, partial *η*^2^ = 0.414. Further analysis for the interaction reflected that, although the inversion effects were similar (*p* = 0.259) between sad and happy conditions (573 ms and 623 ms for upright and inverted happy faces, respectively; *p* < 0.001; 594 ms and 635 ms for upright and inverted sad faces, respectively; *p* < 0.001), quickly happy face classification vs. sad faces was exhibited for upright (21 ms, *p* < 0.005) not for inverted condition (12 ms, *p* = 0.135). In addition, we conducted a Pearson correlation analysis between PCA and the RTs and found that there was an overall significant positive correlation between the RT to negative face stimuli and the size of the PCA, *r* = 0.52, *p* < 0.01 (two tailed), but not between the RT to positive face stimuli and the PCA, *r* = 0.10, *p* > 0.05.

**Table 1:** **The reaction times (RTs) and the amplitudes and latencies of N170 component (across occipital-temporal electrode sites) for upright and inverted face conditions, respectively**.

	Upright faces		Inverted faces	
	Happy	Sad	Happy	Sad
RTs	565 ms	605 ms	615 ms	638 ms
N170 amplitude	−6.4 uV	−7.2 uV	−7.8 uV	−7.8 uV
N170 latency	164 ms	168 ms	173 ms	172 ms

### N170 Component

Grand average ERP waveforms are presented in Figure 2. The effects of the *Expression, Orientation, Hemisphere* and *Site* were analyzed by ANOVA using a mixed model design as described in the “Materials and Methods” Section.

ANOVA of N170 latencies revealed a significant main effect of *Orientation, F*_(1,35)_ = 38.0, *p* < 0.001, partial *η*^2^ = 0.521, with a delayed N170 latency for inverted (172 ms) than upright (166 ms) conditions. The main effects of *Expression* was not significant, *F*_(1,35)_ = 1.41, *p* = 0.24, partial *η*^2^ = 0.039, but qualified by the two-way interaction of *Expression* * *Orientation, F*_(1,35)_ = 9.65, *p* < 0.01; partial *η*^2^ = 0.216. Further analysis for this two-way interaction showed that for upright condition the happy face elicited shorter N170 latency (164 ms) than did the sad faces (168 ms; *p* < 0.02), whereas for inverted condition the N170 latency did not differ between happy (173 ms) and sad faces (172 ms; *p* = 0.20) and that the inversion effect was more conspicuous for happy (inverted *minus* upright: 8 ms) than sad (4 ms; *p* < 0.05) faces. There were no other significant effects (*F*s < 1).

For N170 amplitude analysis, the main effect of *Orientation* was significant, *F*_(1,35)_ = 13.36, *p* < 0.01, partial *η*^2^ = 0.330, showing that overall, face inversion enhanced the N170 amplitudes (−6.8 μV and −7.8 μV for upright and inverted conditions, respectively). The main effect of *Expression* was not significant, *F*_(1,35)_ = 3.37, *p* = 0.075, partial *η*^2^ = 0.088, but the two-way interaction of *Expression* ×*Orientation* was significant, *F*_(1,35)_ = 5.35, *p* < 0.05, partial *η*^2^ = 0.135. Further analysis for this interaction revealed that the effect of *Expression* was evident for upright condition (−6.4 μV and −7.2 μV for happy and sad faces, respectively; *p* < 0.02) not for inverted condition (−7.8 μV and −7.8 μV for happy and sad faces, respectively; *p* = 0.91) and that the effect of *Orientation* was evident for happy (*p* < 0.01) not for sad faces (*p* = 0.08). The main effect of *Hemisphere* was also significant, *F*_(1,35)_ = 8.23, *p* < 0.03, partial *η*^2^ = 0.517, showing the right hemisphere dominance of the N170 amplitude (−6.5 μV and −8.2 μV for left and right hemisphere, respectively). The main effect of *Site* was also significant, *F*_(2,70)_ = 69.23, *p* < 0.001, partial *η*^2^ = 0.589, revealing that the N170 was larger at more occipital-temporal sites (−9.7 μV, −7.8 μV and −5.3 μV for P7/8, O1/2 and TP7/8, respectively). No other effects reached significant level (*p*s > 0.1).

In addition to the ANOVAs, we calculated the Person correlations between the RTs and the amplitude and latency of the N170 for upright face condition (i.e., PCA was evident). While the RTs did not correlate with the amplitude of N170 (*p*s > 0.10), a significant positive correlation between the RTs and the latency of the N170 was found (*r* = 0.47, *p* < 0.05; that is, the longer the RT the longer the N170 latency).

## Discussion

In this experiment we explored the involvement of configural computations while processing positive and negative faces in an expression categorization task. The performance data showed that the classification of happy faces was faster than the classification of sad faces (PCA). Importantly, however, the PCA on the classification speed disappeared for inverted faces. The N170 analysis showed that the N170 was larger for sad than for happy faces only at upright condition and that face inversion significantly enhanced N170 amplitudes only for happy faces. Interestingly, the happy face elicited shorter N170 latency than did the sad faces, whereas for inverted condition the N170 latency did not differ between happy and sad faces. The significant positive correlation between the RTs and the latency of the N170 was also found for N170 amplitudes. Because the configural processing was task-irrelevant in this study, these behavioral and ERP data implicated that the configural analysis is one of the sources of PCA, which is applied by default while categorizing facial emotions.

Several studies have shown the RT advantage for the recognition of happy faces, but none have answered the question whether or not this effect is caused by some high-level configural computations making happy faces visually more distinctive. The present study addressed this question by using upright/inverted schematic happy and sad faces, which were physically comparable but still had the intended emotional value. A widely accepted effect of face inversion refers to the fact that the recognition is severely impaired for inverted relative to upright faces. Actually, the inverted faces impair the structural feature of faces and thus influence configural processing (e.g., Searcy and Bartlett, 1996). Along with this view, the possibly existing difference in configural coding of happy and sad faces is one of sources of the faster categorization of happy faces. Supporting this hypothesis, although face inversion significantly slowed down responses and the inversion effect of RTs is similar between happy and sad faces, the absolute inversion effect of RTs is indeed slightly larger for happy (50 ms) than sad (40 ms) faces, in line with the influence of manipulating configurations larger on happy than sad face identification (e.g., Leppänen and Hietanen, 2004). Supporting this view, Bombari et al. (2013) confirmed that configural processing plays a more prominent role in expression recognition than featural processing, but their relative contribution varies depending on the emotion.There was also evidence that positive and neutral emotions differ to a greater extent than negative and neutral emotions because the configuration of facial features may change more significantly from neutral to happy expression than from neutral to negative emotions (Leppänen and Hietanen, 2004). Moreover, Srinivasan and Gupta (2011) examined the effect of global and local processing on the recognition of sad and happy faces and found that narrowing attention to local processing facilitated the recognition of sad faces, while broad scope of attention facilitated the recognition of happy faces. It should be noteworthy that the above previous study focused on expression recognition, while the present study directly explored the role of configural processing for face classification by expressions.

In the present task, the N170 component was sensitive to emotional expression, as manifested by larger amplitudes to sad than to happy faces. These data support previous findings for early processing of emotional expression (e.g., Caharel et al., 2005; Liu et al., 2013) and suggest that negative emotions engender a more intense emotional reaction than do positive ones. Moreover, converging evidence showed that valence category reflects initial selective attention capture by salient image content (appetitive, threatening) and that unpleasant stimuli can produce stronger emotional effects than can pleasant stimuli—that is, a phenomenon of negativity bias (e.g., Crawford and Cacioppo, 2002). The present findings of enhanced N170 for sad faces is in line with the above view, further indicating that the negativity bias can occur at the early stage of face perception. The present patterns of N170 effects were also consistent with previous findings that valence of affective pictures appeared to influence relatively early (100–250 ms) components of ERPs (for a review, see Olofsson et al., 2008), indicating that affective processing can be described as an automatic feature of perception (e.g., Fox, 1991; Öhman and Soares, 1998). In addition, we found the right-hemisphere dominance of N170 amplitudes, regardless of happy or sad faces. Actually, the well-established right-hemisphere lateralization of the N170 amplitude has been shown in previous studies and this asymmetry is known specifically for faces (e.g., Bentin et al., 1996; Rossion, 2014). Recently, using ERP source-localization techniques Itier et al. (2007) estimated the location of the neural generator of the N170 and found that its neural generators may be located in the fusiform gyrus (FFA), superior temporal sulcus (STS), or both (Itier and Taylor, 2004). However, it should be noted that these techniques are fraught with potential sources of error, and there is disagreement on the validity of inferences drawn from such findings. Therefore, the neutral generators of N170 component await further investigation.

In line with the previous study that the N170 is delayed and enhanced for inverted faces (e.g., Bentin et al., 1996), the present study showed that face inversion enhanced and delayed N170, regardless of facial expressions. However, we found that the inversion effect of N170 was more conspicuous for happy than sad faces. Apparently, this larger N170 inversion effect for happy faces than sad faces further provided electrophysiological evidence for the above hypothesis that the configuration computation was more conspicuous for happy than sad face classification. However, the N170 amplitudes did not correlate with RTs and consequently, the modulation of facial expression on N170 amplitudes did not account for the PCA. In contrast, the happy face elicited shorter N170 latency than did the sad faces, whereas for inverted condition the N170 latency did not differ between happy and sad faces. Importantly, we found a significant positive correlation between the RTs and the latency of the N170. To this end, the present fact of N170 latency implicated that the PCA could be based on high-level configural processing at the early stage of face processing reflected by the face-sensitive N170.

Before concluding, we should reiterate two procedural decisions that constrain the interpretation of the present findings. First, all the faces used in this study were schematic unfamiliar faces to the participants. Using unfamiliar faces we hoped to isolate initial stages of face categorization reducing putative effects of face individuation and identification, processes that might have been tainted by memory factors. Therefore, whether the facial familiarity can modulate the PCA awaits further investigation. Second, in the present study we used schematic faces instead of real faces. Although schematic faces allow us to fully control the low-level physical features, to exclude additional information related to facial identity and to minimize the confounding effects of general arousal rather than valence *per se*, these schematic face pictures are less complex, in particular with respect to the configural components. However, the happiness advantage with schematic facial expressions was consistent with the findings of real faces (e.g., Leppänen and Hietanen, 2004; Liu et al., 2013). Since the happy and sad expressions in the present study equally deviated from neutral faces, it is difficult to attribute the observed advantage of happy faces over sad faces to low-level physical differences between happy and sad faces. In addition, the schematic-face inversion significantly modulated the RTs as well as the N170 component, in line with the face inversion effect of real faces (e.g., Bentin et al., 1996). Hence, the present data further indicate that schematic emotional faces may be ideal experimental stimuli.

In sum, this experiment explored the configural computations while processing positive and negative faces in an expression categorization task. The PCA on the classification speed was evident for upright condition and disappeared for inverted faces. The N170 was larger and delayed for sad faces than happy faces and did not differ for inverted condition. The significant positive correlation between the RTs and the latency of the N170 was also found not for N170 amplitudes. These behavioral and ERP data implicated that the configural analysis could be one of the sources of PCA, which is applied by default while categorizing facial emotions.

## Author Contributions

JS finished experiment and GX finished the article. All authors listed, have made substantial, direct and intellectual contribution to the work, and approved it for publication.

## Conflict of Interest Statement

The authors declare that the research was conducted in the absence of any commercial or financial relationships that could be construed as a potential conflict of interest.
